# Association of Diet With Prostate Specific Antigen and Prostate Volume

**DOI:** 10.5812/numonthly.19411

**Published:** 2014-07-05

**Authors:** Mehdi Shirazi, Ali Ariafar, Shahryar Zeyghami, Mohammad Mehdi Hosseini, Abdol Aziz Khezri

**Affiliations:** 1 Shiraz Institute for Cancer Research, Shiraz University of Medical Sciences, Shiraz, IR Iran; 2 Urology Oncology Research Center, Shiraz University of Medical Sciences, Shiraz, IR Iran

**Keywords:** Prostate-Specific Antigen, Diet, Prostate, Prostatic Hyperplasia

## Abstract

**Background:**

Prostate is an important male reproductive system gland and its disorders can affect men's quality of life and health. Prostatitis, benign prostatic hyperplasia (BPH), and prostate adenocarcinoma are major disorders that can be found in all men in different ages.

**Objectives:**

The aim of this study was to investigate the association of diet with serum prostate specific antigen (PSA) level as well as prostate volume.

**Patients and Methods:**

In this cross-sectional study, 950 men older than 40 years of age who had attended our clinic for a screening program for prostate cancer were enrolled. Data was extracted from the program database. The eligible cases included all noncancerous subjects with available data concerning serum PSA level and prostate volume; the patients had completed a 50-item self-administered food frequency questionnaire about their diet during the preceding two year.

**Results:**

No overall association was found between the consumption of foods and prostate volume as well as serum PSA level. There was a significant correlations between age and serum PSA level (r = 0.24) as well as with prostate volume (r = 0.22) (P < 0.001). In addition, there was a significant correlation between serum PSA level and prostate volume (r = 0.41 and P < 0.001).

**Conclusions:**

The results of this study confirmed the previous reports regarding the serum PSA level correlation with prostate volume. There was no evidence that dietary patterns might have any important effect on prostate volume and serum PSA in this Iranian population.

## 1. Background

Prostate is an important male reproductive system gland and its disorders can affect men's quality of life and health. Prostatitis, benign prostatic hyperplasia (BPH), and prostate adenocarcinoma are major disorders that can be found in all men in different ages. About 75% of men by the age of 70 years develop BPH (1) and histological foci of prostatic intraepithelial neoplasia or adenocarcinoma can be found in most of those over 80 years of age (2). The incidence of prostate cancer has dramatically increased over the last three decades due to the introduction of serum prostate specific antigen (PSA) testing and digital rectal examination. Previous researches have established prostate size as an important predictor of BPH progression (3). Studies indicated that prostatic enlargement increased the risk of acute urinary retention as high as three times the smaller prostates. Therefore, men with prostate volumes larger than 30 cm^3^ had an incidence rate of 4.6% for urinary retention symptoms, whereas this rate was 1.5% in men with smaller prostate (4). Moreover, symptoms are seen three-times more in men with serum PSA level greater than 1.4 ng/mL (5).

In addition to annoying symptoms of BPH, malignancy is a major problem in this field. Several risk factors are confirmed or suspected to be associated with prostate cancer. The inherent factors are age, race, and family history and extrinsic factors include smoking, exposure to sunlight, environmental contaminants, lack of exercise, and dietary pattern. Numerous studies have investigated the role of dietary habits on prostate cancer (6-12); however, a few researches have studied the association of foods consumption with the risk of BPH development (13, 14). Moreover, there is a lack of data about association of food consumption and PSA or prostate volume.

## 2. Objectives

The aim of this study, as the first research on an Iranian population, was to investigate the effect of diet on serum PSA level as well as prostate volume.

## 3. Patients and Methods

This study was a part of a prospective screening program for prostate cancer on 950 men recruited between May 2005 and March 2011 in Shiraz City, Iran. The protocol of study was approved by the Ethics Committee of Shiraz University of Medical Sciences as well as Institutional Review Boards of Shiraz Institute for Cancer Research (ICR) and it was conducted in accordance with the ethical standards set by the Declaration of Helsinki (1975, revised in 1983).

All men older than 40 years of age were invited to participate in the screening program for prostate cancer. Invitation was through brochures, written press releases, and TV announcements. A part of dataset for this study was extracted from the database of the main investigation previously described by Khezri et al. (15). The eligible participants were men who had a PSA test and prostate volume data as well as complete dietary information. The serum PSA level was determined via enzyme-linked immunosorbent assay (ELISA) using a commercial ELISA kit (Can-Ag, Sweden). Transrectal ultrasonography was employed to assess and calculate the prostate volume. The prostate volume was calculated using prolate ellipse formula (volume = width × length × height × 0.52) (16). All ultrasound examinations were performed by one radiologist, using a 6-MHz endorectal transducer.

Dietary data were obtained by means of a 50-item self-administered food frequency questionnaire (FFQ) specifically developed for our study to extract dietary data of the preceding two years. All men who had a previous history of prostate surgery, recent prostate manipulation, prostatitis, a prior diagnosis of prostate cancer, and patients who were already on alpha-blocker or 5-alpha-reductase inhibitor therapy were excluded. Of the 950 records in the database, 803 (84.5%) cases met the eligibility criteria for our study and their data were extracted for further analysis.

Dietary intake was measured by a questionnaire that included food frequency questions; each question was graded on a Likert scale, which were defined as none, less than, equal to, and more than five to ten meals in a month for zero, one, two, and three on the scale, consecutively.

Descriptive statistics including frequency distribution tables and mean ± standard deviations (SD) were generated with the SPSS version 17 (SPSS Inc., Chicago, IL, USA,). Spearman's rank test was used for assessing correlations. The statistical significant level was defined as P < 0.05.

## 4. Results

A total of 803 eligible records were included in our study. The mean age of participants was 59.2 ± 7.3 years (range, 41-88). Table 1 shows levels of PSA and prostate volume in different age groups. The median level of serum PSA in subjects was 0.9 ng/mL (mean, 1.3; range, 0.04-16) and the median volume of prostate was 34 cm^3^ (mean, 37.9; range, 10-173). There were significant correlations between age and both serum PSA level (r = 0.24; P < 0.001) and prostate volume (r = 0.22; P < 0.001). In addition, there was a significant correlation between serum PSA level and prostate volume (r = 0.41, P < 0.001).

The dietary patterns of respondents are shown in Table 2. Amongst the participants, 64.5%, 75.1%, and 60.5% respectively consumed the red meat, chicken meat, and fish meat five to ten times per month. Our FFQ had nine questions about vegetables and fruits including intake of cruciferous vegetables, tomatoes, and other vegetables as well as apples, oranges, cherry, bananas, pomegranate, and nectarine/peaches/apricots. About 75% of men stated no consumption of tomatoes and other vegetables and only 20.5% used cruciferous vegetables of equal to or more than five to ten meals per month. Apples, oranges, and pomegranate were the most frequently used fruits with a rate of 93%, 86.6%, and 84.3% of equal to or more than five to ten meals per month, respectively. In addition, participants were asked about cereals/beans, rice and wheat bread, soy products (commonly texturized soy protein or soy milk), and dairy products. About 65% and 63% respectively used cereals/beans and dairy products of less than five to ten meals per month. About 86% used soy products of equal to or more than five to ten meals per month. After controlling for age, the consumption of food components was related with neither serum PSA level nor prostate volume (Table 3).

**Table 1.** Serum Prostate Specific Antigen Level and Prostate Volume in Different Age groups ^a^

Age, y	No. (%)	PSA, ng/mL	Prostate Volume, cm^3^
**41-50**	72 (8.9)	0.8 ± 0.7	30.9 ± 11.9
**51-60**	429 (53.4)	1.1 ± 1.3	35.3 ± 16.9
**61-70**	250 (31.1)	1.6 ± 1.9	43.6 ± 23.7
**71-80**	45 (5.6)	2.3 ± 2.1	43.2 ± 20.6
**Over 80**	7 (0.9)	3.8 ± 5.3	35.9 ± 10.1
**Total**	803 (100)	1.3 ± 1.7	37.9 ± 19.5

^a^ Data are presented as mean ± SD.

**Table 2.** Food Frequency Data^a^

	Consuming 5-10 Meals/Month			
Foods, No. (%)	None	Less	Equal	More
**Meat**				
Red Meats	6 (0.7)	149 (18.6)	518 (64.5)	130 (16.2)
White Meats				
Chicken Meat	3 (0.4)	65 (8.1)	603 (75.1)	132 (16.4)
Seafood	95 (11.8)	124 (15.4)	486 (60.5)	98 (12.2)
**Vegetables**				
Cruciferous Vegetables	19 (2.4)	619 (77.1)	127 (15.8)	38 (4.7)
Tomatoes	606 (75.5)	22 (2.7)	91 (11.3)	84 (10.5)
Other Vegetables	605 (75.3)	45 (5.6)	124 (15.4)	29 (3.6)
**Fresh Fruits**				
Apples	4 (0.5)	52 (6.5)	348 (43.3)	399 (49.7)
Oranges	6 (0.7)	102 (12.7)	336 (41.8)	359 (44.7)
Cherries	10 (1.2)	306 (38.1)	442 (55)	45 (5.6)
Nectarine/Peaches/Apricots	15 (1.9)	294 (36.6)	453 (56.4)	41 (5.1)
Bananas	8 (1)	289 (36)	451 (56.2)	55 (6.8)
Pomegranates	9 (1.1)	117 (14.6)	458 (57)	219 (27.3)
**Cereals/Beans**	16 (2)	525 (65.4)	252 (31.4)	10 (1.2)
**Rice**	4 (0.5)	91 (11.3)	453 (56.4)	255 (31.8)
**Wheat Bread**	0 (0.0)	26 (3.2)	224 (27.9)	553 (69)
**Soy Products**^b^	4 (0.5)	110 (13.7)	404 (50.3)	285 (35.5)
**Dairy Products**	196 (24.4)	509 (63.4)	97 (12.1)	1 (0.1)

^a^ Data are presented as No. (%).

^b^ Soy products included commonly texturized soy protein or soy milk.

**Table 3.** Correlation of Food Frequency Variables with Serum Prostate Specific Antigen Level and Prostate Volume After Controlling for Age ^a^

	PSA		Prostate Volume	
Foods	r	P Value	r	P Value
**Meat**				
Red Meats	-0.052	0.144	-0.015	0.666
White Meats				
Chicken Meats	-0.072	0.052	-0.024	0.488
Seafood	-0.057	0.107	-0.020	0.564
**Vegetables**				
Cruciferous Vegetables	-0.012	0.726	-0.014	0.700
Tomatoes	0.062	0.081	0.027	0.450
Other Vegetables	0.060	0.088	0.026	0.465
**Fresh Fruits**				
Apples	-0.013	0.716	0.057	0.105
Oranges	-0.060	0.088	0.042	0.238
Cherries	0.033	0.344	0.002	0.960
Nectarine/Peaches/Apricots	0.012	0.733	0.023	0.510
Bananas	0.001	0.984	0.009	0.802
Pomegranates	-0.002	0.952	0.037	0.289
**Cereals/Beans**	-0.026	0.463	0.036	0.308
**Rice**	-0.002	0.952	0.037	0.289
**Wheat Bread**	-0.013	0.716	0.057	0.105
**Soy Products**^b^	-0.016	0.651	0.022	0.529
**Dairy Products**	0.015	0.673	0.041	0.249

^a^ Abbreviations: PSA, prostate specific antigen; and r, correlation coefficient.

^b^ Soy products included commonly texturized soy protein or soy milk.

## 5. Discussion

BPH is one of the most common health problems among old men. Although BPH develops histologically in almost all men by the age of 80 years, its adverse effect on the quality of life can be seen in one-third of men older than 50 years of age. An epidemiologic study on a large Iranian population indicated that about 24% of men over 40 years of age had BPH (17). Despite the considerable effect of BPH and its related morbidity on the public health, its etiology remains unclear; however, several risk factors affect this multifactorial disease such as race/ethnicity, family history, and immunological and endocrinal factors. Among endocrine factors, the role of hormones like androgen, estrogen, growth hormone, and prolactin and growth factors such as insulin-like growth factors, fibroblast growth factor, and transforming growth factors have been recognized previously (14).

Our cross-sectional study was conducted on a large population in a screening program for prostate cancer and investigated the association of dietary patterns on prostate size and serum PSA levels. There are numerous studies that investigated the association of food consumption and prostate disorders such as BPH or cancer.

A case-control study in Greece was performed by Lagiou et al. on 184 patients with BPH and 246 controls (18). Nutrient intakes for individuals were estimated by a validated semi-quantitative FFQ through multiplying the nutrient contents of a selected typical portion for each specified food item by the frequency of that food consumption; then these estimates were summed for all food items. The questions were about dietary intakes during the preceding year. Thereafter, protein, total fat, saturated, monounsaturated, and polyunsaturated fat, carbohydrates, and dietary fiber intakes in grams and total energy in kilocalories were estimated. In addition, daily micronutrient intakes, e.g. sodium and potassium, were calculated in milligrams. They indicated that risk of BPH was increased with added lipids, butter, and margarine and was decreased with fruit intake. Bravi et al. conducted a study on 1369 patients with BPH and 1451 controls in Italy and investigated the usual diet of participants during the previous two years through a FFQ. They found a significant increasing risk of BPH with more frequent consumption of cereals, bread, eggs, and poultry as well as a decreasing risk with soups, pulses, cooked vegetables, and citrus fruit. They observed no association between BPH and dairy products, coffee and tea, pasta and rice, fish, cheese, row vegetables, potatoes, fruit, or desserts (19). Ambrosini et al. performed a case-control study on 406 and 462 Australian men (age range, 40-75 years) with and without BPH, respectively, and investigated the association of BPH with usual dietary intake during the preceding ten years (20). They used a semi-quantitative FFQ to collect data on dietary intake that listed 74 foods or food groups, each with ten intake frequency choices ranging from "never" to "three or more times per day". Then the completed FFQs were analyzed to provide intakes in grams per day for each FFQ item. They stated that BPH development was positively correlated with consuming high-fat dairy foods and negatively with consumption of vegetables, soy products, and red meat. There are contradictory results in different studies; some studies showed a decreased risk of BPH with limited consumption of red meat and fat, high intake of protein and vegetables, and regular alcohol drinking (21, 22). With regard to the correlation between diet and PSA, Ohwaki et al. showed a negative association between PSA and protein intake and a positive association between fat intake and PSA levels (23).

Some other studies assessed the association of prostate cancer with diet. Hodge et al. in Australia found that foods rich in olive oil, tomatoes, and allium vegetables might reduce the risk of prostate adenocarcinoma (24). In a recent study, Ax et al. revealed that low-carbohydrate high-protein diet is inversely associated with prostate cancer incidence (25). On the other hands, some investigators like Muller et al. in Melbourne Collaborative Cohort Study found no association between any dietary pattern and prostate cancer by assessing 17045 men (26). Similarly, Takachi et al. studied 321061 Japanese men and observed no association between prostate cancer and consumption of fruits and vegetables (27). According to our study, there was no evidence that diet could influence the serum PSA level or prostate sized. We also found a direct association between age and serum PSA level as well as prostate volume. These findings are also in accordance with previous studies in the United States and Saudi Arabia (28, 29). Similarly, Safarinejad’s study on Iranian population demonstrated a strong correlation (r = 24) between prostate volume and age (17).

Our study had some limitations such as inability to calculate the exact quantitative amount of consumed food in our FFQ for each participant and lack of a validated FFQ during conducting this study; however, available FFQs had some potential limitations (30) including individual’s memory, accuracy of estimations, and nutrient database precision. FFQs are the only feasible and most commonly used method in assessment of past dietary intake; however, they are based on self-report and interviews, are associated with biases and potential errors, and their validity highly depends on study members' understanding as well as the list of food items un the questionnaire. Although FFQs are not considered suitable for estimating true dietary intake at the individual level, their application is recommended for epidemiological studies to rank individuals along the distribution of intake and to discriminate the low-intake individuals from high-intake ones (31). Thus, vegetables were not consumed monthly by three out of four cases in our study or dairy products were consumed daily by less than 0.1% of the sample.

Another limitation to our study was the limited period of recording dietary data, i.e. during the preceding two years. This might not include the food habits and changes during or prior to this period. On the other hand, diet can indirectly affect the prostate characteristics by chronic changes in metabolic syndrome variables, which could not be reflected in a two-year FFQ.

This study showed that total dietary intakes were not associated with the increased serum PSA level or prostate size. In addition, we found age as a strong factor for elevation of serum PSA level and prostatic enlargement; however, further cohort studies can provide more valuable results.
